# Mechanoresponsive musculoskeletal tissue differentiation of adipose-derived stem cells

**DOI:** 10.1186/s12938-016-0150-9

**Published:** 2016-04-22

**Authors:** Andrew Trumbull, Gayathri Subramanian, Eda Yildirim-Ayan

**Affiliations:** Department of Bioengineering, College of Engineering, University of Toledo, Toledo, OH 43606 USA; Department of Orthopaedic Surgery, University of Toledo Medical Center, Toledo, OH 43614 USA

**Keywords:** Adipose-derived stem cells, Musculoskeletal tissue, Differentiation, Mechanical strain, Biomechanics, Mechanobiology, Bone, Tendon, Cartilage, Muscle, Ligaments, Tissue engineering

## Abstract

Musculoskeletal tissues are constantly under mechanical strains within their microenvironment. Yet, little is understood about the effect of in vivo mechanical milieu strains on cell development and function. Thus, this review article outlines the in vivo mechanical environment of bone, muscle, cartilage, tendon, and ligaments, and tabulates the mechanical strain and stress in these tissues during physiological condition, vigorous, and moderate activities. This review article further discusses the principles of mechanical loading platforms to create physiologically relevant mechanical milieu in vitro for musculoskeletal tissue regeneration. A special emphasis is placed on adipose-derived stem cells (ADSCs) as an emerging valuable tool for regenerative musculoskeletal tissue engineering, as they are easily isolated, expanded, and able to differentiate into any musculoskeletal tissue. Finally, it highlights the current state-of-the art in ADSCs-guided musculoskeletal tissue regeneration under mechanical loading.

## Background

Musculoskeletal disorders such as osteoporosis, osteoarthritis, tendon and ligament tears along with bone defects represent an increased major health problem worldwide [1]. Around the world, disabilities due to these musculoskeletal disorders have increased by 45 % in last 10 years and are expected to further increase with an increasingly obese, sedentary, and ageing population [2, 3]. Musculoskeletal disorders affect millions of people from all ages in all cultures and ethnicities. Current estimates of people affected worldwide from back pain is 632.045 million, while neck pain affects 332.049 million, osteoarthritis knee affects 250.785 million, and other musculoskeletal conditions impact 560.978 million people [4]. Autografting and allografting are still considered to be the golden standards in repairing musculoskeletal defects; however, they inherently have limited availability, require an additional surgical procedure with potential complications, and are susceptible to immunorejection [5]. Stem cell transplantation and stem cell/scaffold-guided tissue engineering approaches are emerging as viable alternatives to grafting.

Stem cells are rapidly emerging as an invaluable resource in musculoskeletal tissue engineering because they can greatly enhance the regenerative capabilities of engineered grafts through their unique properties [6]. Stem cells incorporated in tissue defects have been shown to promote regeneration in in vivo neural [7, 8], cartilage [9, 10], bone [9, 11], tendon [12], and muscular applications [13, 14]. To this end, extensive research is being conducted on embryonic stem cells (ESCs), induced pluripotent stem cells (iPS), and adult stem cells utilization for musculoskeletal tissue engineering. While embryonic and induced pluripotent cells have unlimited differentiation capacity and close to infinite replication potential, they are currently limited in their applications [6, 15]. Ethical concerns greatly limit ESC-based therapies [6], and iPSs may have genetic abnormalities and issues with tumorigenicity, which present major concerns for their therapeutic applications [16, 17]. Utilizing adult stem cells -specifically adipose-derived stem cells (ADSCs)—in musculoskeletal tissue regeneration is an ideal alternative to ESCs and iPSs, as they are easy to harvest without ethical concerns, abundant within the body, and compatible with patient’s immune system.

Stem cell-based regenerative therapy, however, is still in its infancy for musculoskeletal tissue regeneration. One emerging approach to increase the effectiveness of stem cells’ differentiation in musculoskeletal applications is to apply mechanical strain to the cells in vitro [18]. Applying mechanical strain to the cells in vitro helps us to predict the cells’ in vivo behavior, and devise treatments to maximize their regenerative potential. In a similar manner, before implantation, cells may be exposed to dynamic mechanical strain in vitro to differentiate and prepare them for the in vivo mechanical environment. The success towards this direction depends on understanding the specific mechanical milieu around different musculoskeletal tissues and developing relevant in vitro platforms to closely mimic in vivo mechanical conditions.

In vivo, musculoskeletal tissues are under a myriad of mechanical loading modalities, which include compression, stretching, and fluid flow. For instance, bones are primarily under compression loading, however, within the bone tissue osteocytes are under fluid flow stress [19]. Muscles normally experience tensile stretch in vivo [20, 21], while cartilage tissue is primarily subjected to compressive loads and hydrostatic pressure, but can experience shear at the surface layer as well [22]. Thus, in a musculoskeletal tissue engineering approach, an in-depth look into a musculoskeletal tissue’s physiology and mechanical environment is necessary to create physiologically relevant mechanical loading platforms for cells to differentiate into functional musculoskeletal tissue.

This comprehensive review paper is composed of three major sections. In the “Background” section, the mechanical environments around major musculoskeletal tissues including bone, cartilage, tendon, ligament, and muscle are elucidated. The mechanical loading modalities and mechanical strain values exerted on these tissues are further tabulated. In “The mechanical environment of musculoskeletal tissues” section, the principles behind various mechanical loading modalities which represent in vivo mechanical loading conditions including compression, tensile (uniaxial and biaxial stretching), and fluid flow are discussed. Representative in vitro studies, which applied various mechanical loading techniques to musculoskeletal tissue associated cells, are discussed in this section as well. In the “Principles in mechanical loading platform design for musculoskeletal applications” section, the advantages of utilizing adipose-derived stem cells in musculoskeletal research over other stem cells are discussed. Furthermore, the in vitro studies which have investigated the differentiation potential of ADSCs towards musculoskeletal tissue associated cells such as chondrocytes, osteoblasts, tenocytes, myocytes, and ligament-like cells under different mechanical strains and frequencies are discussed. The results from these representative in vitro studies are further compiled and tabulated based on mechanical loading modality and targeted tissue. In this table, mechanical loading parameters including strain magnitude, loading frequency, and duration were provided along with the origin of the cells used in these studies and the effects of mechanical loading on cells. It should be also noted that, with this review we do not attempt to provide a detailed review on these mechanical loading platforms because this has been the focus of excellent reviews in the literature [23–29].

## The mechanical environment of musculoskeletal tissues

There is an overwhelming amount of evidence suggesting that the mechanical environment around musculoskeletal tissues affects the regeneration and degeneration of those tissues depending on the mechanical loading type and magnitude. Since different musculoskeletal tissues are exposed to different types of in vivo mechanical strain, each musculoskeletal tissue’s mechanobiological niche must be taken into consideration when designing mechanical loading platforms.

### Mechanical environment around bone tissue

**Fig. 1:**
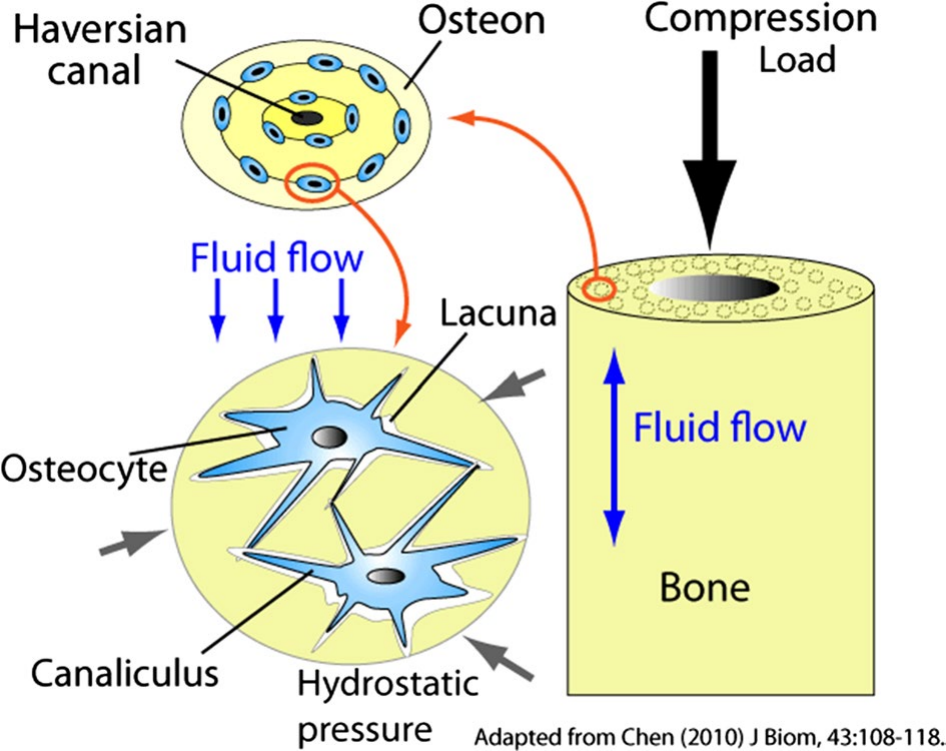
Schematic illustration of bone tissue under compressive and fluid flow loading. Adapted from [40]

Bone, as musculoskeletal tissue, is responsible for transmitting forces applied by muscles to enable locomotion and mechanical action of limbs with little deformation. In mechanically active regions of bones, the mineralized matrix is composed of parallel cylinders of dense vascularized bone called osteons. These osteons are made of concentric sheets of aligned and mineralized collagen fibers, with subsequent layers having varied collagen orientations from 0 to 90° [30]. As bones transmit and resist forces during a myriad of vigorous activities, many different force types are acting on whole bones; compression, bending, tensile, shear, torsion, and biaxial forces are all relevant to bone mechanics [31]. Bone cells experience compression and tensile strain due to forces from muscles and outside sources during locomotion, which deform the whole bone [32–36]. Shear forces arise due to interstitial fluid flow displaced during bone deformation around osteocytes contained in hollow canals called lacuna within the mineralized bone [31, 37–39]. Figure 1 demonstrates the translation of forces on a whole tissue level into fluid shear on a cellular level.

Several research groups managed to quantify the deformations experienced by bone during low level physiological loading, hemostatic physiological loading, and vigorous physiological activity [41–44]. The deformations in bones are expressed as mechanical strain (ε), where 1000 µε equals a 0.1 % change in the length of the bone as compared to its original length [31, 45]. In daily life, bones routinely experience mechanical strains between 1000 and 2000 µε [46–48]. In vivo studies demonstrated that mechanical strains below 1000 µε create catabolic effects on bone remodeling [45, 49] and strains above 4000 µε initiate fracture within the bone [46], while mechanical strains around 3000 µε generated by vigorous exercise stimulate new bone formation. In extreme circumstances, such as falling on outstretched arms, strains can reach 6000 µε, or as much as 9096 µε during basketball rebounding [50]. Even though in vivo bone strain ranges from 0.1 to 0.5 %, in vitro studies have demonstrated that osteoblasts and osteocytes do not display detectable biochemical responses to strains below 0.5 % [46, 51, 52]. In fact, in in vitro studies, researchers generally have to apply above 1 % strain on bone cells to detect certain changes in osteogenesis [46]. This discrepancy in in vitro and in vivo strain levels would suggest that in vivo, the mechanical strain is amplified during the strain transfer from the extracellular matrix to bone cells. This amplification in vivo is generally attributed to complex interactions between the uniquely structured bone matrix and osteocyte cell processes [46, 53]. Due to the comparatively simplified nature of in vitro mechanical strain devices, it is therefore necessary, and commonly accepted, to apply increased strain levels of around 1–10 % on cultured cells.

### Mechanical environment around tendon and ligament

The primary role of tendons is to transmit forces from muscle to bone in order to produce body movement while ensuring joint stability. Tendons in vivo thus operate in a very dynamic environment and are known to withstand huge mechanical forces. The distinct biomechanical properties of tendons that allows them to bear large loads is attributed to the high degree of organization of the tendon extracellular matrix (ECM) [54]. The ECM of tendons is mainly composed of parallel bundles of collagen type-1 fibers arranged in a hierarchical fashion in a proteoglycan matrix. Highly aligned collagen fibers within the tendon tissue play a major role in supporting and transmitting the uniaxial tensile forces generated by muscle contraction to bones [54, 55].

Tendons are viscoelastic in nature and the collagen fibers have a characteristic ‘crimp pattern’ structure that enhances their mechanical behavior during loading [56]. Various factors, however, affect the level of in vivo mechanical strains experienced by tendons. Different tendons are known to experience different levels of strains depending on their location in the body. Larger cross-sectional areas in muscles produce larger forces which results in higher strains on tendons [57]. Significantly, tendons have the unique ability to undergo tissue mechanical adaptation in response to loading. It is well-established that exercise and mobilization of tendons result in increases in their cross-sectional areas and hence their tensile strength [58, 59], while immobilization of tendons decreases their modulus [60, 61]. Furthermore, varying the level of physical activity can result in different strain levels on the same tendon. Since tendons have viscoelastic characteristics, their response is affected by the rate and frequency of loading [62, 63]. Tendons are found to be more deformable at lower strain rates, and possess a higher degree of stiffness at larger strain rates [64].

**Fig. 2:**
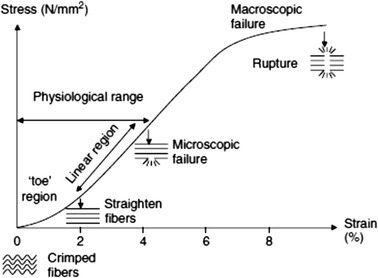
A typical stress–strain curve for tendons showing defined toe, linear and failure regions [57]

On being subjected to mechanical forces, a typical tendon stress–strain curve exhibits three distinct phases, as shown in Fig. 2 [57]. The curve has an initial toe region when strained up to 2 %, during which the ‘crimp pattern’ of the tendon stretches out to produce normal locomotion. In the case of increased physical activity that produces strains in the range of 2–4 %, the collagen fibers lose the ‘crimp pattern’ due to which the loaded tendon behaves purely elastically, as depicted by the linear region of the curve. Generally, the physiological loading of human tendons occurs within the strain defined by the toe and linear regions, beyond which the tendon is more susceptible to injuries. The material properties have been determined for many tendons such as Achilles [65, 66], patellar [67, 68] and tibialis anterior tendons [69] across different mammalian species including humans in terms of modulus and failure stress–strain values. Due to the diversity of tendons and their ability to adapt functionally, the values fit in a broad range. Young’s modulus in the range of 500–2000 MPa and failure stress between 50–150 MPa have been generally observed. Strains at complete rupture range from 5–16 % for mid-tendons and up to 32 % for tendon-bone insertion sites [65, 70]. Microscopic tearing of tendon fiber is observed at 4–6 % strains, that progresses to macroscopic failure at 8–10 %, followed by ultimate tendon rupture [55, 57].

Ligaments possess a similar structure to tendons; they are composed of bundles of collagen fibers, with cells sparsely distributed along the fiber bundles. The main function of ligaments -which distinguishes them from tendons- is that they connect two bones, and serve to stabilize them, especially in joints. Ligaments’ primary biomechanical role is to resist uniaxial tensile stress, as evidenced by their aligned morphology. However, as they are involved in joint stabilization, and wrap around moving bones, shear, transverse, and compressive loading are also biologically relevant [71].

### Mechanical environment around muscle

Muscle can experience both uniaxial, biaxial, and shear strains depending on which muscle is being studied; skeletal, cardiac, or smooth. Skeletal muscle cells are 10–100 μm thick, several centimeters long, and organized in parallel bundles. As evidenced by their unidirectional arrangement, skeletal muscle experiences uniaxial strain, and its primary function is to linearly contract to transfer force to bones. Smooth muscle on the other hand, lines organs and blood vessels, and must expand often in either uniaxial or biaxial directions to accommodate enlargement of tissues [21]. It can also experience shear stress from blood flow penetrating past the thin layer of endothelial cells in the blood vessel lumen [72]. Just as mechanical loading modalities for muscle change depending on type, strain levels also vary depending on location in the body. Vascular smooth muscles around blood vessels experience approximately 5 % strain due to swelling of the vessels [73], and even as much as 9–12 % in the aorta during systole [74]. Skeletal muscles experience much more stress, reaching as much as 9 KN or 12.5 times bodyweight during vigorous exercise [75], a 5–9 % change in length during moderate physical activities such as walking [76], and up to 12 % during sprinting [77].

### Mechanical environment around cartilage

Cartilage tissue in the musculoskeletal system is primarily responsible for cushioning and providing low-friction articulation in joints. Collagen content within the cartilage provides the tensile and shear strength to the tissue, while the water -held in the tissue by its attraction to proteoglycans- absorbs over 90 % of compressive load transmission [78]. The cartilage structure is divided into three main regions. The superficial zone makes up about 10 % of the total volume and is composed of tightly compacted collagen and chondrocytes arranged parallel to the surface, and give this zone exceptional tensile strength. The middle zone comprises about 60 % of the total volume and consists of obliquely organized collagen fibers and few chondrocytes, which give it a higher compressive modulus than the superficial layer. The deep zone makes up 30 % of the total thickness and has many vertically arranged collagen fibers, which give it the lowest water content and highest compressive modulus [79, 80].

In vivo, the magnitude of contact stresses exerted on cartilage can range from 1 to 6 MPa under vigorous physical activities and can reach to as high as 12 MPa depending on activity type and duration [81]. It should be noted that contact stresses on cartilage are always compressive. The compressive strain on cartilage can be calculated by dividing the applied cartilage stress value by the compressive modulus of cartilage tabulated in the literature [81]. Due to the anisotropic structure of cartilage, compressive strain values vary greatly within cartilage tissue [22]. While the superficial zone experiences over 50 % compressive strain, the middle and deep zones experience only 0–5 % compressive strain [22, 82]. For overall cartilage tissue, in vivo studies demonstrated that the total thickness of cartilage may decrease anywhere from 0–5 % under continuous static loading [82]. In vivo knee MRI data collected before and after exercises showed that continuous static loads of 150 % body weight caused only a 3 % femoral cartilage thickness loss after 1 min of moderate exercise [83]. Under the physiological loading frequencies (0.01–2 Hz) of cartilage, the thickness of patella–femoral cartilage decreased up to 5 % after vigorous exercise [84]. Besides, compressive forces, shear forces are also exerted on cartilage-bone interface, which range from 2 to 5 % with a maximum value at superficial zone and decrease to negligible values at the deeper zones [82]. Table 1 summarizes the strain or stress magnitudes experienced by musculoskeletal tissues in vivo.

**Table 1:** In vivo mechanical strain and stress in major musculoskeletal tissues

Tissue type	In vivo mechanical loading	In vivo strain or stress magnitudes	Ref
Bone	Fluid shear stress	0.8–3.0 N/m^2^	[165, 166]
	Tensile strain	0.03–0.1 % (though close to10 % is widely accepted to account for in vivo amplification)	[165]
Tendon	Tensile strain	2–4 % (physiological range)	[57]
	Tensile strain	5–16 % (complete rupture for mid-tendon)	[66]
Skeletal muscle	Tensile stress	9 kN (vigorous exercise)	[75]
	Tensile strain	5-12 % (moderate activity)	
Cartilage	Compressive stress	1–6 MPa (vigorous activity)	[81]
	Compressive strain	50 % (at superficial zone)	[62]
	Compressive Strain	0–5 % (at deep and middle zone)	[63]

## Principles in mechanical loading platform design for musculoskeletal applications

Musculoskeletal tissues are constantly under mechanical strains within their microenvironment [46, 85, 86]. Numerous studies have been conducted toward understanding the greater role of microenvironmental factors on cell behavior [86, 87]. However, still little is understood about the effect of in vivo mechanical milieu strains on cell development and function because of the complexity of cellular systems and the difficulty in isolating the solo effect of mechanical strains from other extracellular factors [46, 88]. In order to conduct systematic studies to understand how mechanical strains around musculoskeletal tissues play a role in cell behavior, a concerted effort has been made toward creating tools to capture physiological mechanical strains in vitro. Numerous types of mechanical loading platforms have been employed to create physiologically relevant mechanical strains on cells and cell-encapsulated scaffolds to regenerate musculoskeletal tissues. The choice of the mechanical loading platform is dependent on which musculoskeletal tissue or associated cells are being studied and what types of mechanical loading the tissue experiences in vivo. Three common mechanical loading modes affiliated with musculoskeletal tissues are compression, stretching, and fluid flow, all with various strain levels and frequency. These loading modalities are applied to stem cells and stem cell-embedded tissue scaffolds using mechanical loading platforms, which were the focuses of excellent reviews in the literature [23–29].

### Compression

As musculoskeletal tissues, bone and cartilage are primarily under compression loading, as we discussed in the previous section. To simulate the compression loading milieu around these tissues in vitro, compressive mechanical loading is applied following two methods: hydrostatic compression of cells and platen compression of cell-embedded tissue scaffolds.

*Hydrostatic compression* systems apply strain to cells by increasing the pressure of the media the cells are in. In order to apply compression to the cells, they are either seeded in monolayer [89] or in three-dimensional (3D) scaffolds [90] and submerged in media. Commonly, the chamber containing the cells does not contain a gas layer as a pressure increase in the presence of a gas may alter media pH or composition [91]. Hydrostatic pressure is generally applied by compressing a cylinder to increase the pressure of the fluid in the chamber, or directly compressing the culture chamber. Therefore, the strain on the cells is not measured in percent strain, but rather in applied stress, in MPa. One advantage of hydrostatic compression is the ease of application and modulation of hydrostatic pressure; unlike other methods, the strain is applied directly to the cells and not indirectly through a scaffold or other medium. Hydrostatic compression has been applied to monolayer cells to determine their gene expression under in vivo loading conditions [92], to fabricated 3D constructs to monitor new extracellular matrix secretion [93], or to cartilage explants to more closely model in vivo conditions [94].

**Table 2:** Adipose-derived stem cells studies conducted under various mechanical loading

Mechanical loading modality	Targeted tissue	Mechanical loading parameters (modality, strain and frequency, and duration)	Cell origin	Effect	References
Uniaxial stretch	Osteoblasts	Uniaxial 2D four-point stretch 0.24 % strain applied for 2 h for 5–10 days	Human	Osteogenic gene profile ↑ Mineral deposition ↑	[145]
		Uniaxial 2D four-point stretch 0.2 % strain with 1 Hz frequency for either 17 min/day for 10 days or 6 h for 1 day	Rat	BMP-2 and RUNX2 (after 6 h loading) ↑	[146]
		Uniaxial 3D stretch 10 % strain with 1 Hz frequency 4 h/day for 14 days	Human	Paladin gene expression ↑	[147]
		Uniaxial 2D four-point stretch 0.2 % strain with 0.5 Hz frequency 2 h/day for 7 days	Human	Osteogenic gene expression and ALP activity were increased up to day 5 but were decreased at day 7	[144]
		Uniaxial 2D flexcell tension system 10 % strain with continuous 1 Hz or rest-inserted (1 Hz with 10 s between cycles) frequency 4 h/day for 14 days.	Human	Calcium deposition ↑ both loading groups	[148]
		Uniaxial 2D clamp stretch 5 % strain with 1 Hz frequency 15, 60, and 120 min single and triplicate applications	Human	Osteogenic gene expression ↑ all strain regimes except single 15 min	[149]
		Uniaxial 3D clamp stretch 10 % strain with 1 Hz frequency 4 h	Human	Calcium deposition ↑ Osteogenic gene profile ↑	[150]
Fluid flow		Pulsatile 2D fluid shear 0.6 Pa mean shear 0.3 Pa pulse amplitude 8.4 Pa/s peak shear applied at 5 Hz frequency for 1 h	Human	NO production ↑ Osteogenic gene expression ↑	[143]
Uniaxial stretch	Tenocytes	Uniaxial 3D stretch 4 % strain with 2 h stretch followed by 6 h rest cyclically applied for 21 days	Equine	Tenogenic morphology ↑ Tenogenic gene expression ↑	[160]
Uniaxial stretch	Myocytes	Uniaxial 2D flexcell system 11 % strain with 0.5 Hz 1 h/day for 19 days	Human	Myogenic gene expression ↑, multinucleation ↑, myotubes ↑	[153]
		Uniaxial 2D stretch across post 10 % strain with 1 Hz frequency for 7 days (continuous)	Human	Cell alignment ↑	[113]
		Uniaxial 2D stretch across pistons 15 % strain with 0.5 Hz frequency for 48 h	Human	Fusion with myoblasts ↑ Cell alignment ↑	[154]
		Uniaxial 2D stretch 10 % strain with 1 Hz frequency for 24 h	Rat	Myogenic gene profile ↑	[155]
		Uniaxial 2D flexcell stretch 12 % strain with 1 Hz frequency for 48 h	Human	No significant positive effects	[152]
		Uniaxial 2D stretch 5 % strain with 1 Hz frequency for 14 days	Human	Smooth muscle differentiation markers ↑	[156]
Biaxial and uniaxial stretch		Equiaxial 2D stretch over post 10 % strain with 1 Hz frequency for 24 h	Rabbit	Myogenic gene GATA4 expression ↑	[151]
Compression	Chondrocytes	Cyclic 3D platen compression 5 % strain with 1 Hz frequency 4 h/day for 7 days	Rabbit	Calcium signaling pathways ↑ proliferation ↑ Aggrecan production ↑, collagen production ↑	[98]
		Cyclic 3D hydrostatic compression 10 MPa pressure applied with 1 Hz frequency 4 h/day 5 days/ week, 5 weeks	Porcine	Glycosaminoglycan content ↑	[158]
		Cyclic 3D hydrostatic compression 0.4 or 5 MPa pressure applied with 0.5 Hz frequency for 4 h/day, 5 day/week for 4 weeks	Human	Glycosaminoglycan content (both groups) ↑ Collagen II, aggrecan, and sox-9 gene expression (both groups) ↑	[90]
		Cyclic 3D hydrostatic compression 7.5 MPa applied with 1 Hz frequency 4 h/day for 7–21 days	Human	Chondrogenic gene profile day 7 ↑ Gene expression and viability at days 14 and 21 ↓	[159]

*Platen compression* utilizes a plate or platform to directly compress a specimen, and is usually employed to compress 3D tissues or cell-embedded tissue scaffolds. Unlike hydrostatic compression, however, the strain is applied to a 3D construct in which the cells are seeded, and therefore the strain may be measured in percent strain or applied stress. Platen compression is used in many cellular applications including determining cellular responses for explants from different regions of cartilage [95], and increasing graft strength through increased cell activity [96]. Platen compression may be carried out using commercially available hydraulic servos or linear actuators [95, 97] to deliver specific strains or stresses to constructs in custom compression chambers, with commercial compression bioreactors such as the Bose BioDynamic ELF5110 [98], or with other custom made devices [99].

### Uniaxial and biaxial stretching

#### Uniaxial stretch

**Fig. 3:**
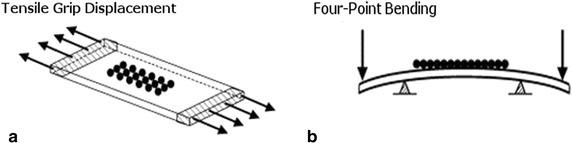
Schematic illustrating techniques for longitudinal stretch application including: **a** uniaxial tension via grip system resulting in longitudinal displacement, and **b** membrane bending caused by applied mechanical stimulus, either a load or displacement. Adapted from [28]

Muscle, tendon, and ligament are under tensile stretching, as we discussed in the previous section. To mimic the mechanical milieu around these tissues, uniaxial mechanical loading platforms apply uniaxial stretching to either monolayer cells attached to deformable membranes or directly to cell-embedded tissue constructs. Uniaxial stretching, also known as longitudinal stretching, is applied to membrane or tissue constructs in a single direction through gripping at either end of the membrane and applying uniaxial tension (Fig. 3a). Uniaxial stretch can also be achieved using four-point or substrate bending, which employs controlled bending of deformable membranes to apply the desired uniaxial stretch (Fig. 3b).

The amount of strain transferred to a construct by tensile grip is determined simply enough by measuring the displacement of the grips. Determining the amount of strain transferred to a construct through four-point loading, however, is more difficult as it depends on multiple variables. The equation for determining uniaxial strain from a four-point loading device is

$$\varepsilon = \frac{6Fa}{{bh^{2} E}}$$

where F is the force applied, a is the distance from force applied to support post, b is the width of the substrate, h is the length of the substrate, and E is the elastic modulus [100].

One advantage of uniaxial mechanical loading platforms is the ease of viewing such systems under a microscope at any time during strain application. This enables the user real-time analysis of effects due to mechanical loading. One consequence of using uniaxial systems is that they may produce strain gradients perpendicular to the direction of stretch. This allows investigation into the effect of strain gradients on cell behavior. While the capability of these uniaxial stretch devices to impose strain gradients can be advantageous, it can also increase the difficulty of data interpretation when unintended. In this respect, biaxial stretch may be preferred, as it can provide relatively homogenous strain gradients.

#### Biaxial stretch

In contrast to uniaxial stretch, which allows stretch in only one direction, biaxial stretch provides multi-directional stretch both longitudinally and laterally or radially and circumferentially (for circular membranes). Biaxial stretch can be divided into two categories: out-of-plane stretch and in-plane stretch.

*Out*-*of*-*plane stretch* is the deformation of a membrane away from the neutral axis, which can be achieved using platen displacement, prong displacement, vacuum distension, and fluid displacement. The platen displacement occurs by deforming the flexible membrane in an upward direction, which causes tensile biaxial strain. The prong displacement utilizes a vacuum to distend the deformable membrane over the prong or post placed below, exposing the adherent cells to tensile strain. In vacuum distension technique, membranes can be distended downward using pure vacuum suction. This method results in compressive strain at the center and tensional strain at the periphery, due to the deformation achieved upon suction. The fluid displacement technique utilizes fluid to deform the membrane in upward direction. Numerous studies applied out-of-plane stretch to investigate its effect on cells associated with musculoskeletal tissues. While out-of-plane mechanical strain provides a simple way to apply biaxial stretch, it too, exhibits strain gradients. This leads to cells in different areas of the membrane experiencing different strain, which can make it difficult to discern the effect of such forces on cell behavior. Additionally, as this stretch occurs outside of the focal plane, it can be difficult to complete real-time imaging of the adherent cells during strain cycles. While imaging can be supplemented with computer based programming to focus the area of interest, the use of such technology is not common.

**Fig. 4:**
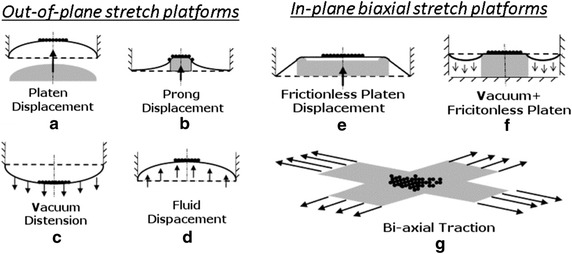
Schematic illustrating techniques for out-of-plane biaxial stretch application including: **a** membrane deformation via platen displacement, **b** prong displacement, **c** vacuum distension, and **d** upward deformation via fluid displacement and for in-plane biaxial stretch application including **e** membrane deformation via frictionless platen displacement, **f** vacuum distension over a frictionless platen, and **g** stretch via bi-axial traction. Adapted from [28].

*In*-*plane biaxial stretch* was developed to address the heterogeneity of applied strain and to provide homogeneous strain to adherent cells. Three methods used to create *in*-*plane biaxial stretch* are frictionless platen displacement, vacuum frictionless platen displacement, and biaxial traction. Figure 4 illustrates the schematic of out-of-plane stretch and in-plane biaxial stretch platforms. In frictionless platen displacement method, cells are quarantined to a particular zone to create homogenous strain. The vacuum frictionless platen displacement method utilizes vacuum suction to cause uniform membrane stretch over a frictionless platen, while in biaxial traction methods, mechanical stretches are applied both in longitudinal and lateral axes. All of these methods theoretically provide equi-biaxial strain at the membrane center. One assumption associated with such systems is that the surfaces are deemed frictionless, negating boundary influences. In-plane biaxial strain provides uniform strain distribution to adherent cells, allowing for clear understanding of cell response to applied mechanical stimulation. However, movement of the cellular membrane can cause disturbance to the liquid medium layer. As with platen compression, this movement within the system can result in unintended shear and pressure forces, possibly affecting cell response to stretch.

In mechanobiology research, commercially-available and custom-designed mechanical loading platforms are used. The most popular commercially-available units are Flexcell International Cooperation (Burlington, NC). The company manufactures mechanical loading platforms applying various mechanical strains to monolayer culture or 3D cell culture. Mechanical loading stations from Flexcell provide uniform radial and circumferential strains to cells cultured on flexible membranes. Vacuum is applied to the flexible membrane to deform the membrane across the post creating equibiaxial strain. Kariya et al. [101] used a Flexcell unit to apply biaxial strain to murine osteoprogenitor cells to understand the mechanism of mechanically-induced differentiation. They measured ATP activity of mechanically-stretched cells and unloaded cells and they concluded that mechanical stretched increased the ATP activity of cells, which caused an increase in intracellular Ca + 2 concentration that resulted in enhanced osteogenic differentiation. Ignatius et al. [102] used a custom-built apparatus and applied uniaxial strain to the silicone plate where an osteoblast cell-encapsulated collagen matrix was deposited onto. Their results demonstrated that uniaxial strain increased the osteogenic activity of human osteoblasts within the collagen matrix compared to unloaded counterparts. Kao et al. [103] utilized cyclic uniaxial loading to murine osteoprogenitor cells and showed that cyclic loading enhanced osteogenic differentiation of these cells significantly. Wang et al. [104] also investigated the mechanically-induced osteogenic differentiation through applying biaxial strain to osteoprogenitor cells. Besides osteoblast and osteoprogenitor cells, other musculoskeletal tissue related cells were studied under uniaxial and biaxial loading. For instance, Ahearne et al. [105] studied tissue-engineered tendon using uniaxial loading to strain the cell-embedded collagen scaffold. Their results showered that uniaxial strain induced alignment in collagen fibers and tenogenic activities. Ralphs et al. [106] also investigated how actin stress fibers and cell-cell adhesion molecules in tenocytes responded to the uniaxial mechanical loading. Scientists have also studied the ligament cells under uniaxial loading. Pan et al. [107] studied ligament cells under cyclic uniaxial strain to identify the role of cyclic strain on cytoskeletal rearrangement. Kang et al. [108] and his group utilized uniaxial strain to differentiate human umbilical cord-derived mesenchymal stem cells into ligament-like cells. Similar tensile loading applications have been used in the field on mesenchymal stem cells [109, 110], embryonic stem cells [111], and adipose-derived stem cells [112, 113] to induce their differentiation to the specific musculoskeletal lineages. Other cell types that have been studied under either biaxial or uniaxial strain include chondrocytes [114], tenocytes [105, 106, 115], ligament cells [107, 108], and myoblasts [116].

### Fluid flow

**Fig. 5:**
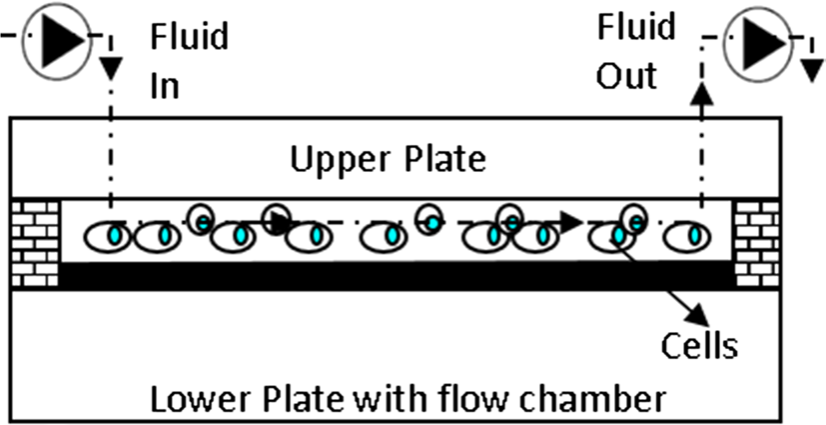
Schematic view of fluid flow chamber with cells. Adapted from [117]

Among musculoskeletal tissues, osteocytes in bone tissue are the primary cell type subjected to shear stress created by fluid flow. Thus, mechanical loading of cells using fluid flow is a major tool for studies primarily focused on osteocytes and the role of fluid shear in bone hemostasis. Fluid flow mechanical loading platforms generate well-defined shear stresses to cells using viscometers and flow chambers. In viscometer platforms two configurations have been used, namely the cone-and-plate configuration and parallel plate configuration. For cone-and-plate viscometer, the shear stress can be calculated by

$$\tau = \mu \frac{w}{\alpha }$$

where µ is the viscosity of the fluid, α is the angle that cone makes with stationary plate, and w is angular velocity of the rotating plate. For parallel plate viscometer, the shear stress can be calculated by

$$\tau = \mu \frac{w r}{h}$$

where r is the radial position of the cells from the center of the plate and h is the height between the plates [100]. As seen in above equation shear stress on cells varies from 0 (at the center of the plate) to a maximum at the edge of the plate. Similar to the tensile types of mechanical loading, this creates nonhomogeneous stress distribution across the cultured cells, which causes complications in interpreting cellular data affected by the shear stress. Flow chamber configuration may eliminate this issue by providing fully developed, constant laminar flow. In flow chamber systems, cells are seeded in a rectangular chamber created by two parallel plates. The fluid is driven through the chamber by a pump to create shear stress. Figure 5 demonstrates the schematic of a fluid flow chamber with cells.

A fluid flow with low Reynolds number (Re < 2) should be created within the chamber to ensure laminar flow. The flow creates shear stress on cells and the chamber wall. It is assumed that the shear stress on the chamber wall (τ) is equal to shear stress on the surface of the cells. It should be noted that this assumption is valid if the width of the chamber (b) is much bigger than the height of the chamber (h) (b ≫ h). The shear stress exerted on cells can be calculated by

$$\tau = \frac{6Q\mu }{{bh^{2} }}$$

Fluid flow shear is generally applied to bone or endothelial cells, to mimic the in vivo environments of blood vessels and interstitial fluid flow in bone. Riddle et al. [118] applied oscillatory fluid flow on MSCs in a custom parallel plate flow chamber to increase the osteogenic activity of MSCs. Djien Tan et al. [119] used a custom parallel plate flow chamber to instead modulate osteoclast activity. Breen et al. [120] developed a custom cone and plate viscometer to apply fluid shear to endothelial cells.

## Adipose-derived stem cells differentiation towards musculoskeletal tissues under mechanical loading

### Why adipose-derived stem cells?

Adult stem cells can be harvested from many sites such as lung tissue, umbilical cord blood, bone marrow, adipose tissue, and synovial tissue. These cells have been the focus of much regenerative research in the musculoskeletal tissue engineering field. The two types of adult stem cells that have been most utilized in tissue engineering are bone marrow-derived mesenchymal stem cells (BMSCs) and adipose-derived mesenchymal stem cells (ADSCs) [121]. BMSCs are generally harvested through piercing into bone using a syringe, and aspirating a fraction of bone marrow [122]. Bone marrow aspiration, however, creates great discomfort for the donor and may lead to chronic pain along with possible donor site morbidity [123].

ADSCs are a great alternative to BMSCs due to their ease of harvest and nearly identical differentiation potential [124]. ADSCs are extracted during a minimally invasive liposuction procedure [125, 126], which is less painful and causes less donor site morbidity compared to bone marrow aspiration [124]. Furthermore, the number of isolated stem cells from adipose tissue is higher than bone marrow. In bone marrow aspirate, BMSCs compose 0.002 % of cells present in the marrow aspirate, while ADSCs are about 2 % of the cell population in adipose tissue [127, 128]. ADSCs have proven their merit as a valuable tool for regenerative musculoskeletal engineering. Preliminary clinical studies, in vitro, and in vivo studies demonstrated that ADSCs can be differentiated into a desired cell lineage, and utilized in regenerative therapies. For instance, Thesleff et al. successfully used ADSCs to repair cranial defects and saw restoration to normal cranial integrity [129], and Mesimaki et al. had success repairing a maxillary defect using autologous ADSCs expanded in vitro [130]. Yoshimura et al. [131] showed that ADSCs used in breast augmentation provided results that were superior to conventional lipoinjection.

The lineage commitment of ADSCs is usually controlled though soluble differentiation factors incorporated into the media or scaffold. However, it has been well proven that structural, chemical, and mechanical cues also affect the ADSCs differentiation and lineage commitment. For instance, Betre et al. [132] showed that elastin-like polypeptide hydrogels promoted chondrogenesis in ADSCs in the absence of differentiating factors. Flynn et al. [133] found that ADSCs cultured in non-adhesive hydrogels differentiated into adipocytes faster than ADSCs that were cultured in an adherent placenta-derived matrix, or a mixture of the two. Tseng et al. [134] showed that scaffold alignment caused cells to exhibit a tenocyte-like morphology. Rada et al. [135] showed that culturing ADSCs on hydrogel and gelatin scaffolds with differing mechanical properties significantly affected the cells expression of chondrogenic genes, morphology, and proliferation. Furthermore, it has been shown that physiologically relevant strain application can also affect the differentiation of ADSCs. Below is the concise review of the studies concentrated on mechanical strain-induced differentiation of ADSCs into various musculoskeletal tissue associated cell lineages.

### Specific lineage commitment of ADSC under mechanical strain

Adipose stem cells have been used in multiple in vivo studies and have proven their value as regenerative tools; ADSCs have been used in vivo to repair bone [130, 136–138], muscle [13, 14, 139], cartilage [10, 140–142], and tendon [12]. Only one of these studies, however, investigated the effects of strain on the effectiveness of the cells. Knag et al. [142] used a rotary bioreactor to apply some fluid stress to the cells before implantation, but the investigators did not note specifics on strain levels or rate, and somewhat attributed the noted increased regenerative potential to the more rapid nutrient exchange caused by the flowing media. These in vivo studies, especially the one by Mesimaki et al. [130] which used ADSCs to repair a maxillary bone defect in a human patient, indicate that ADSCs are soon to become an irreplaceable clinical tool. However, due to the relative difficulty of in vivo strain measurement and characterization, in vitro studies such as the following will be invaluable in in vivo application when understanding the effects of physiological strain.

#### Differentiation into osteogenic lineage

The differentiation of ADSCs towards an osteogenic lineage is the most-studied out of all the musculoskeletal lineages, however research is still preliminary. The following section outlines the currently completed studies on the osteogenic response of ADSCs to mechanical stimulation. Out of the following studies, all used uniaxial strain except a study by Tjabringa et al. [143] which utilized pulsitile fluid flow. All studies used a 1 Hz strain rate except for the study by Du et al. [144] which utilized a 0.5 Hz rate. In accordance with the previous section describing in vivo bone mechanobiology, many of the studies applied strains of 10 % which is above the commonly accepted whole bone levels of 0.1–0.2 %, but nonetheless acceptable as stated above. Three studies used strain levels of ~0.2 %, however, and saw significant increases in the osteogenic activity of the cells. This indicates that ADSCs are responsive to a large range of applied strains, including the low levels seen acting on whole bones in vivo. The precise effects of different strain, however, have not yet been determined, and therefore the following reports vary in application, though no evidence has been conclusive.

Ye et al. [145] studied the osteogenic differentiation of ADSCs under uniaxial loading. Human ADSCs and BMSCs (hADSCs and hBMSCs) were seeded on a polyethylene disk in osteogenic medium, and were subjected to tensile strain once they reached 80–90 % confluence for a total differentiation time of 14 days. A four-point bending device was used to apply strain on the magnitude of 0.24 % (2400 µε) at a frequency of 1 Hz with loading occurring for 2 h every day. ALP, mineralization, and PCR for RUNX2, BMP-2, ALP, and Osteocalcin (OC) were analyzed to determine osteogenic differentiation of hADSCs and hBMSCs. Strain significantly increased the mineral deposition and expression of ALP, BMP-2, and RUNX2 at 5 and 10 days in hADSCs. OC was only increased at day 10. However, hBMSCs had significantly more mineralization, and expression of BMP-2 at days 5 and 10 and ALP at day 10. This study showed that hBMSCs have a greater ability to differentiate into an osteogenic lineage than hADSCs. However, this study also showed that mechanically dynamic culture has a great effect on the differentiation of hADSCs towards an osteogenic lineage.

Yang et al. [146] studied the differentiation of ADSCs toward an osteogenic lineage under sustained or repeated uniaxial strain. ADSCs were harvested from rats, and osteoinduced in osteogenic media for 48 h before loading. Cells were maintained in osteogenic media, and stretched for either 10 days straight for 17 min or just 1 day for 6 h. All cells were stretched at 1 Hz and 0.2 % strain using uniaxial 4-point bending. Measurements were taken 2 h after end of last loading session. PCR for ALP, Osteocalcin (OCN), BMP-2, and RUNX-2 was performed and results showed upregulation (though not significant) of BMP-2 for day 7, and a slight peak for OCN at day 7. Only significant measurements were BMP-2 and RUNX2 for 6 h group. Cell alignment was observed for day 10, but not 6 h.

Wall et al. [147] studied the osteogenic differentiation of human ADSCs towards an osteogenic lineage under prolonged uniaxial strain. ADSCs were seeded into 70 % collagen gels and subjected to 14 days of 10 % uniaxial strain at 1 Hz for 4 h a day. This study showed that paladin—a gene that is upregulated as ADSCs differentiate towards an osteogenic lineage—was upregulated in response to cyclic strain.

Du et al. [144] cultured human ADSCs in differentiaion media with and without uniaxial cyclic strain (groups MC and C, respectively) to determine the effect loading had on the osteogenic differentiation of ADSCs. hADSCs were cultured in normal conditions for 48 h, then cultured in osteogenic media in static or strained culture. Cells experienced uniaxial strain by a 4-point bending apparatus of 0.2 % (2000 µε) at 0.5 Hz frequency 2 h a day, with a culture time of 7 days. ALP activity in group C induced saw a steady increase starting at day 3 and continuing to day 7. In group MC, ALP activity began to increase at day 2, but peaked at day 5 and day 7 saw a significant decrease. Gene expression of ALPL, COL 1, RUNX2, SPP1, and SPARC, as analyzed by PCR followed the exact same trends seen in ALP activity for both groups. On days 2, 3, and 5 group MC had higher expression of all genes and ALP, but on day 7, expression/activity dropped close to group C. This showed that mechanical strain can be useful for osteogenic differentiation of hADSCs along with chemical signals, but that the effects may decrease with time. Their results suggested that the decrease of differentiation signals may be due to aging of cells due to prolonged loading.

Tjabringa et al. [143] studied osteogenic differentiation of ADSCs under pulsatile fluid shear stress. hADSCs were cultured on polylysine coated glass slides and cultured overnight, the next day they were exposed to a 5 Hz pulsatile fluid flow (PFF) with a mean shear stress of 0.6 Pa, a pulse amplitude of 0.3 Pa, and a peak shear stress of 8.4 Pa/second. Samples of the media were taken at 0, 10, 30, and 60 min to measure NO production. PCR was run examining COX-1, COX-2, Runx-2, and osteopontin (OP) at 3 and 6 h after PFF. It was found that PFF significantly increased NO production at 60 min of PFF. PFF did not affect OP or COX-1, but increased COX-2 by 6- and 5-fold after 3 and 6 h respectively, and Runx2 1.3-fold at 3 h. These results prove that hADSCs have a bone cell-like response to mechanical loading even in the absence of chemical induction factors.

Hanson et al. [148] observed the osteogenic differentiation of two lines of ADSCs with differeing osteogenic potential under uniaxial strain. Two groups of hADSCs were initially assessed for deposition of calcium on tissue culture plastic in differentiation medium. One cell line showed approx. nine times as much calcium deposition as the other. The cells were then exposed to cyclic strain, and their proliferation, viability, and calcium deposition was measured again. The cells were seeded in bioflex culture plates and cultured for 5 days, until they reached 100 % confluence. Then 10 % uniaxial strain of 1 Hz and 4 h a day was applied to the cells for 2 weeks using a Flexcell tension plus system. Cells were either subjected to continuous 1 Hz strain, or rest-inserted strain (10 s between each cycle). No difference was shown between either the continuous or rest-inserted strain groups. Loading of either type increased calcium deposition, though the increase was significantly greater in the cell line with higher initial deposition on static culture plastic.

Diederichs et al. [149] observed the osteogenic differentiation of ADSCs under different lengths and repetitions of uniaxial strain regimes. Different lengths of application of strain were applied to hADSCs, ALP activity and expression of early and late osteogenic markers was investigated. Cell viability and collagen III expression (expressed under excessive strain) were observed to see if strain was excessive. Cells were induced in osteogenic differentiation medium on collagen I coated silicone plates for a week before application of uniaxial strain. Afterwards, cells had 15 min, 60 min, and 2 h strains applied to them at 1 Hz and 5 % by clamping and stretching of the silicone plates. Additionally, triple strain regimes were carried out with double the strain rest inserted e.g. 15 min strain–30 min rest–15 min strain–30 min rest–15 min strain. Cells were immediately harvested after application of strain. ALP activity was slightly decreased in repeated 15 min but increased in both 1 and 2 h repeated. Both the elongated and repeated strain were effective at enhancing differentiation as evidenced by upregulation of osteogenic genes, but repeated was always better than elongated at strain acclimation.

Charoenpanich et al. [150] measured osteogenic gene expression and calcium accretion of hADSCs was measured under the influence of mechanical strain, 3D culture, and osteogenic media. 24 h after seeding in 70 % collagen gels, cells were put in growth or osteogenic (50 mM ascorbic acid, 0.1 mM dexamethasone, and 10 mM b-glycerophosphate) media. Cells were loaded with 10 % strain at 1 Hz for 4 h/day in a stretching anchored collagen gel, after which microarray analysis was performed to determine gene upregulation. Mechanical strain was shown to increase the expression of PDLIM4 which is one of the top five upregulated genes shown to have polymorphisms often found with bone mineral density. Calcium accretion that was increased in the loaded cells was found to be controlled by different genetic pathways than soluble chemical factors. Additionally, 10 % strain resulted in upregulation of two crucial factors in bone regulation; proinflammatory cytokine regulators IL1RN and SOCS3, and angiogenic inducing factors FGF2, MMP2, and VEGF A. 3D culture also seems to use a different pathway than 2 days for osteogenic induction

#### Differentiation into myogenic lineage

Multiple studies have shown that ADSCs have potential in myogenic tissue engineering, and that mechanical strain can have a significant impact on their differentiation into muscle cells. The strain applied in the following experiments ranged from 5 to 15 %, with five studies applying uniaxial strain, and one applying biaxial strain. Strain was generally applied for 24 h a day for anywhere from 1 to 14 days, except one study where strain was applied for 1 h a day for 21 days. Only one study, by Amin et al. [151] tested for myogenic activity under strain without chemical induction, and found that mechanical strain was enough to significantly increase the myogenic activity of the ADSCs, though not as much as strain with chemical induction. Multiple studies showed that mechanical loading also enhanced the fusion of ADSCs with myocytes to form muscle fibers. Conversely, studies by Dugan et al. [152] and Lee et al. [113] showed that did not have a positive myogenic effect on the ADSCs. While not all the studies showed that mechanical strain aids in the differentiation of ADSCs, the majority indicate that mechanical strain can be a powerful tool for differentiation of ADSCs, especially along with chemical induction.

As previously stated, Amin et al. [151] studied the effect that equiaxial strain had on the differentiation of ADSCs. GATA4, a transcription factor that plays an important role in late embryonic heart development, was measured as a determinant of myogenic differentiation. Cells were seeded on 2D collagen membranes and exposed to 5-azacytidine for 24 h, then 10 % equiaxial cyclic strain was applied at 1 Hz for 24 h after removal of differentiating medium. Cells were cultured for 1, 4, and 7 days before assays were performed. GATA4 was upregulated most at day one by only mechanically stimulated cells, but for days 4 and 7—while still greater than control- it was surpassed by mechanically strained and chemically induced group.

Dugan et al. [152] studied the effects of cyclic uniaxial strain on ADSCs cultured with C2C12 myoblasts in myogenic media. Murine C2C12 myocytes and Human ADSCs were seeded on Flexcell flexible-bottom 6-well plates in a ratio of 1:5 and cultured for 24 h before myogenic media was added, and strain was applied. 12 % strain was applied continuously for 48 h at 1 Hz in pulses with 1 s duration and 1 s rest inserted between each application. After strain was applied, cells were cultured under static conditions for 5 days before being analyzed. However, after analyzing myogenic activity of the ADSCs using immunocytochemistry and gene expression, it was determined that strain had no apparent effect on the ADSCs myogenic activity either in monoculture, or in co-culture with C2C12 cells.

Lee et al. [113] studied effects that TGF-β_1_ and uniaxial strain had on the differentiation of ADSCs into smooth muscle. Cells were plated on collagen coated flexible plates, and cultured for 3 days before uniaxial mechanical strain by membrane stretching across a loading post. Cells experienced continuous 10 % strain, at 1 Hz for 7 days. Additionally, cells were cultured with TGF-β_1_ to induce smooth muscle differentiation. Cell proliferation was nearly identical in all cultures, and while TGF-β_1_ increased smooth muscle actin and calponin expression, strain did not appear to positively affect the differentiation of the cells. Strain did, however, align the cells on the plates.

Yilgor et al. [153] studied the effect that chemical induction and uniaxial strain had on human ADSC differentiation into a myogenic lineage. ADSCs were induced with myogenic induction medium 24 h after seeding on flexible membranes, after another 24 h, cells were washed and, media was replaced with growth media. Cells were exposed to 11 % uniaxial strain at 0.5 Hz for an hour a day during days 3–21 of culture using a Flexcell system. It was found that chemical induction was necessary for multi-nucleation of cells, as well as expression of muscle specific protein (desmin, myoD, and myosin heavy chain). Desmin and myoD were significantly greater in the dynamic culture conditions, unlike the static conditions. Myotube count and nuclei/myotube were significantly higher in dynamic culture.

Andersen et al. [154] studied the effects myogenic media, uniaxial strain, and co-culture with mouse myoblasts had on the myogenic differentiation of ADSCs. Cells were allowed to reach 90–95 % confluence on flexible-bottom plates coated with collagen with and without mouse myoblasts (with a ratio of 1:5). Mechanical 15 % uniaxial strain was applied at 0.5 Hz using rectangular pistons for 48 h in myogenic media. After stimulation, cells were cultured in differentiation media for differing amounts of time. ADSCs were infected with viral particles including GFP, to aid in monitoring ADSC differentiation. The culture conditions did not appear to increase the differentiation of ADSCs towards a myogenic lineage; however, it did greatly increase their rate of fusion with myoblasts and their alignment.

Bayati et al. [155] observed the effects of uniaxial loading on ADSCs with and without differentiation media. Gene expression and myosin synthesis was monitored in rat ADSCs after 3 days of chemical differentiation with 5-azacytidine, and loading on day 2. Cells were seeded on collagen-1 coated silicone membranes and cultured for 48 h, before addition of differentiation media. Addition of differentiation media occurred on day 1, and 10 % 1 Hz 24 h uniaxial loading was performed on day 2 using a clamping tensile device. The results showed that MyoD, myh2, and myog were significantly increased under strain in both control and myogenic differentiation media.

Park et al. [156] observed the myogenic differentiation of hADSCs exposed to many differentiation factors, along with uniaxial strain. Cells were seeded on PLCL scaffolds 3 days prior to stimulation, and then put in induction media, and stretched at 5 % at 1 Hz for 2 weeks using a uniaxial clamp device. Strain greatly increased the expression of a-SMA and MHC in certain myogenic media (retinoic acid, ascorbic acid, “smooth muscle induction media”) therefore, this study indicates that strain can be very beneficial to the induction of hADSCs to a myogenic lineage.

#### Differentiation into chondrogenic lineage

Few studies have been done on the role of mechanical loading on ADSC differentiation into chondrocytes. On the other hand, the ability of ADSCs to differentiate into a chondrogenic lineage through the use of chemical factors has been confirmed by many studies [151, 155, 157]. Out of the four studies that were found, only one used platen compression, while the other three utilized hydrostatic compression. The peak amplitudes of hydrostatic pressure applied ranged from 5 to 10 MPa, while 5 % compressive strain was applied under platen compression. All studies applied strain for 4 h a day and strain was applied from 7 to 35 days. These results show that under physiological loading conditions, ADSCs will differentiate effectively into chondrocytes even at longer time points.

Li et al. [98] studied the chondrogenic differentiation of rabbit ADSCs exposed to dynamic platen compression and insulin-like growth factor 1 (IGF-1) in chitosan/gelatin scaffolds. Cells were cultured in the scaffold for 2 days, and then they were subjected to 5 % compressive strain at 1 Hz for 4 h per day for 7 days. Compression loading was carried out by placing scaffolds between two platens in a compression bioreactor (BioDynamic ELF5110). Dynamic compression alone upregulated calcium signaling pathways and Sox-9 (which is known to induce chondrogenesis in BMSCs).

Carroll et al. [158] applied cyclic hydrostatic pressure to porcine ADSCs in agarose scaffolds to induce their chondrogenic differentiation. ADSCs were seeded in 2 % agarose gels, sealed in water-tight bags with 4 mL medium per construct. The bags were then placed in a water filled bioreactor which exposed them to 1 Hz cyclic hydrostatic pressure for 4 h a day, 5 days a week for 5 weeks, with a peak amplitude of 10 MPa. Sulfonated Glycosaminoglycan (sGAG) content, which is a key structural component of cartilage, was significantly increased in constructs exposed to cyclic hydrostatic pressure, which in turn increased the dynamic and equilibrium modulus of the constructs. Additionally, it was shown that when a chondrogenic growth factor (TGF-β3) was removed in some constructs after the first week, the loaded samples were able to retain collagen and sGAG content better than unloaded samples. Altogether, this study demonstrated the positive effects of hydrostatic pressure on both inducing and maintaining chondrogenic differentiation in ADSCs.

Correia et al. [90] investigated the effects that high and low cyclic hydrostatic pressure had on the differentiation of human ADSCs into a chondrogenic lineage. ADSCs encapsulated in gellan gum hydrogels and suspended in chondrogenic media were exposed to two different strain regimes as well as a static, unloaded control. The first group was exposed to 0.5 Hz cyclic pressure with a peak amplitude of 0.4 MPa for 4 h a day, 5 days a week for 3 weeks; the second was exposed at 0.5 Hz with a peak amplitude of 5 MPa for an identical length. The group exposed to 5 MPa pressure showed most GAG content from staining (Safranin O and Alcian Blue), while the 0.4 MPa group showed less than the 5 MPa group, but significantly more than the control group. However, gene expression of collagen II, aggrecan, and sox-9 indicated that 0.4 MPa was the most effective, though 5 MPa was still improved over control. These results show that ADSCs in vitro sense and respond in a chondrogenic-positive manner to cyclic hydrostatic pressure.

Puetzer et al. [159] showed that cyclic hydrostatic pressure even in the absence of chondrogenic differentiation factors was able to induce chondrogenic differentiation in ADSCs. ADSCs were encapsulated in 2 % agarose hydrogels, and exposed to cyclic pressure at 1 Hz with a peak amplitude of 7.5 MPa for 4 h a day for up to 21 days. At day 7, loaded cells exhibited better chondrogenic gene profiles as compared to control groups. However, at days 14 and 21, cells exhibited lower viability and decreased chondrogenic gene expression. This study indicated that cyclic loading can initiate chondrogenic differentiation of ADSCs even in the absence of chondrogenic differentiation factors.

#### Differentiation into tenogenic lineage

At the time of the writing of this article only one publication has shown the effects of mechanical loading on the tenogenic differentiation of ADSCs. In the study, Raabe, O. et al. exposed equine ADSCs were exposed to 21 % oxygen tension, as well as differentiation factors, and tensile strain and measured cell morphology and expression of tendon-relevant genes (collagen type 1 and 3, cartilage oligomeric protein, and scleraxis). Cells were seeded in collagen gels, and then compacted under 2.7 and 5.2 kPa around for 4 h until desired thickness was achieved. Cells were then put in a uniaxial linear stretching device for 21 days with 4 % strain for 2 h followed by 6 h rest. Authors concluded that differentiation of ADSCs in collagen seems to be best carried out with strain, and factors as evidenced by cell morphology and gene expression [160]. Many other studies have also shown that ADSCs have the ability to differentiate into tenocytes with differentiation medium [161], and that strain assists in the differentiation of BMSCs into tenocytes [25, 27]. Therefore, further investigations into positive effects of strain on the tenogenic activity of ADSCs should be further studied.

#### Differentiation into adipogenic lineage

In musculoskeletal tissue engineering, it is important to make sure that cells seeded in scaffolds differentiate into the desired type. A major concern when using ADSCs is that they may differentiate into adipocytes. Confirming that ADSCs do not exhibit an adipogenic phenotype is crucial to the integrity of the implant site. Recent research has shown that mechanical loading can inhibit the differentiation of ADSCs into adipocytes, making it an even more attractive tool in musculoskeletal engineering.

Yang et al. [162] observed the adipogenic differentiation of adipogenically induced murine ADSCs Murine ADSCs were adipogenically induced for 3 days, then seeded on polyethylene plates, and after 6 h adipogenic media was replaced. Mechanical loading of 0.2 % (2000 µε) at 1 Hz for 2 or 6 h began immediately using a four point bending apparatus. ADSCs that had not been adipogenically-inducted underwent the same loading conditions. Loading significantly reduced the amount of oil droplet filled cells visible. Additionally, both 2 and 6 h significantly decreased PPAR-γ and increased Runx2 and Pref-1 in osteogenically induced cultures, as compared to their static counterparts. Control ADSCs, however, did not show a significant difference in gene expression compared to static un-induced when exposed to 2 or 6 h of strain. These results indicate that mechanical strain can hinder adipogenic differentiation, and that osteogenic and adipogenic lineages may be inversely related.

Huang et al. [163] seeded ADSCs on collagen-coated flexible bottom plates and allowed to attach for 16 h. Mechanical strain was then applied using a Flexcell 4000T tensile system with 0.5, 2, or 10 % at 0.5 Hz for 48 h in adipogenic or control media. This study found that 10 % loading applied to old mouse ADSCs increased their proliferative potential, and that 2 and 10 % strain levels inhibited adipogenic differentiation.

Li, G., et al. [164] exposed ADSCs to adipogenic media for 3 days, then seeded onto loading plates, after 6 h adipogenic media was added, and cells were subjected to 4-point mechanical loading of 0.2 % (2000 µε) at 1 Hz for 2 or 6 h. Oil red O was used to asses fat droplet formation 2 h after end of loading, and adipogenic genes, PPAR-c1 (a critical transactivator of adipogenesis) and APN (one of the adipocyte-predominant proteins), were examined by real-time PCR. Oil red O showed a significant reduction in fat droplet deposition in the 2 h loading compared to control, and in the 6 h group compared to the 2 h group. Both APN and PPAR-γ expression were significantly decreased for 2 and 6 h; APN was reduced to 80 % and PPAR-γ to 50 % for 2 h, and APN decreased to 20 % and PPAR-γ to 3 % for 6. This study found that mechanical compression significantly reduced the adipogenic differentiation of ADSCs even in adipogenic differentiation media.

In addition to studies that show mechanical strain as a useful tool in the differentiation of ADSCs into various musculoskeletal lineages, the results of these studies show that it can also inhibit adipogenesis. These results further solidify the need for mechanical loading in the musculoskeletal tissue engineering field. Table 2 compiles the results of representative studies utilized ADSCs along with various mechanical loading modalities to differentiate them into different linages.

## Conclusion

In recent years, adipose-derived stem cells (ADSCs) have been considered to be powerful tools for musculoskeletal tissue engineering. The easy and minimally invasive access to adipose tissue makes adipose-derived stem cells a superior alternative to bone-marrow derived mesenchymal stem cells. Furthermore, compared to mesenchymal stem cells, ADSCs have equal differentiation potential when considering cells of mesodermal origin such as cartilage, bone, tendon, and skeletal muscle. Studies investigating the effects of mechanical loading on ADSCs differentiation solidify the importance of the cells mechanical environment on its musculoskeletal lineage commitment. Understanding physiological levels of mechanical strains experienced by musculoskeletal tissues is essential to create physiologically relevant mechanical loading platforms used to differentiate ADSCs. To this end, we first attempted to compile information about the mechanical strains experienced by musculoskeletal tissues in the body and how this information can be utilized to create mechanical loading platforms for in vitro studies. Finally, we introduced the current state-of-the-art in the differentiation of adipose-derived stem cells using these mechanical loading platforms and various physiologically relevant mechanical loading modalities. A major challenge, however, will be the translation of mechanically-induced ADSC differentiation concepts into clinical settings to regenerate damaged musculoskeletal tissues. Fundamentally, future research in the area of ADSC mechanobiology-based tissue engineering needs to incorporate both experimental and theoretical methodologies to identify how mechanical loading is transferred from a musculoskeletal tissue scaffold to the individual cells and how this loading is translated in intracellular and extracellular cell components. Focusing on these challenging concepts will transform the ADSC mechanobiology-based tissue engineering field and will help move bench-top research into clinical applications.
